# Supplementary material for: Identification of photoperiod-induced specific miRNAs in the adrenal glands of Sunite sheep (Ovis aries)
Source: Front Vet Sci. 2022 Jul 22;9:888207. doi: 10.3389/fvets.2022.888207 (PMC9354845; doi:10.3389/fvets.2022.888207)
Supplement: Supplementary file 1 [file Data_Sheet_1.ZIP › Supplementary file/miRDeep2 file.zip!/miRDeep2 file/Know_Structure/LP42A3/oar-mir-655.pdf]

miRBase precursor : oar-mir-655
Total read count : 3950
oar-miR-655-5p read count : 14
oar-miR-655-3p read count : 3935
remaining reads : 1

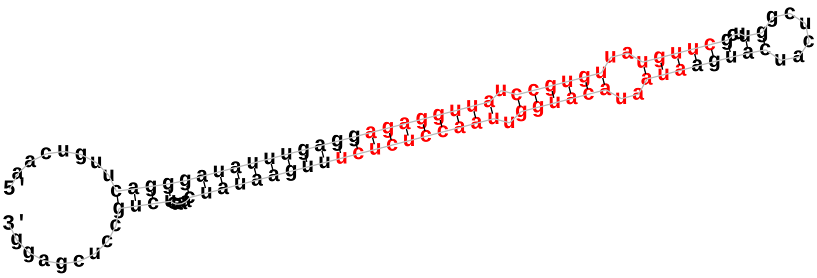

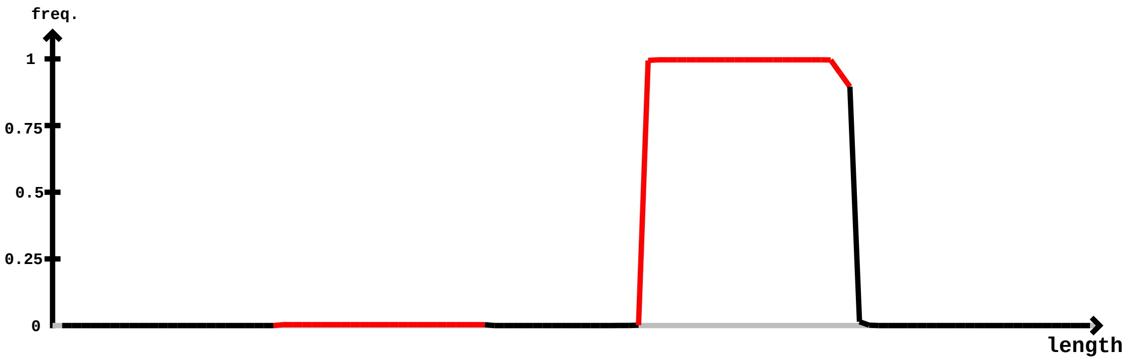


oar-miR-655-5p

oar-miR-655-3p

| | | | |
|---|---|---|---|
| 5'- aacuguucagggauauuugaggagagguuauccguguuauguucgcuuggcucaucaugaauaauacaugguuaaccucucuuugaauaucaaacucugccucgagg -3' | exp | | |
| ...........(((((((((((((((((((.((((((..((((((..((......))))))))..)))))).)))))))))))))))))))...(((......))). | reads | mm | sample |
| ......................agagguuauccguguuauguu................................................................ | 1 | 0 | seq |
| ......................agagguuauccguguuauguuc............................................................... | 13 | 0 | seq |
| .........................................................ugaauaauacaugguuaaccucu........................... | 1 | 0 | seq |
| ...........................................................aauaauacaugguuaaccucu........................... | 3 | 0 | seq |
| ...........................................................aauaauacaugguuaaccucucu......................... | 2 | 0 | seq |
| ............................................................auaauacaugguuaaccuc............................ | 2 | 0 | seq |
| ............................................................auaauacaugguuaaccucu........................... | 195 | 0 | seq |
| ............................................................auaauacaugguuaaccucuc.......................... | 197 | 0 | seq |
| ............................................................auaauacaugguuaaccucucu......................... | 3473 | 0 | seq |
| ............................................................auaauacaugguuaaccucucuu........................ | 48 | 0 | seq |
| ............................................................auaauacaugguuaaccucucuuu....................... | 6 | 0 | seq |
| .............................................................uaauacaugguuaaccucucu......................... | 6 | 0 | seq |
| .............................................................uaauacaugguuaaccucucuu........................ | 3 | 0 | seq |
